# Operationalizing African self-reliance in vaccine manufacturing

**DOI:** 10.1080/16549716.2025.2560209

**Published:** 2025-10-03

**Authors:** Chiluba Mwila, Anna Mia Ekström, Beate Kampmann, Stefan Swartling Peterson, Nicholas Alexander, Nebiyu Dereje, Abebe Genetu Bayih, Jean Kaseya, Ole Petter Ottersen

**Affiliations:** a Africa Centres for Disease Control and Prevention, Addis Ababa, Ethiopia; b Department of Global Public Health, Karolinska Institutet, Stockholm, Sweden; c Department of Infectious Diseases/Venhälsan, South General Hospital, Stockholm, Sweden; d Charité Center for Global Health, Charité-Universitätsmedizin Berlin, Berlin, Germany; e Makerere University School of Public Health, Kampala, Uganda; f Sustainable Health Unit (SUSTAINIT), University of Oslo, Oslo, Norway

**Keywords:** vaccine production, Africa CDC, African Pooled Procurement Mechanism (APPM), Framework for Action (FFA), Platform for Harmonized African Health Products Manufacturing (PHAHM)

## Abstract

The COVID-19 pandemic underscored Africa’s urgent need for vaccine security and self-reliance. In response, the African Union and Africa Centers for Disease Control and Prevention (CDC) established the Framework for Action (FFA) through the Platform for Harmonized African Health Products Manufacturing (PHAHM), with a goal of 60% local vaccine production by 2040. During 2024, Africa CDC, Karolinska Institutet, and Charité Universitätsmedizin Berlin organized a seminar series to discuss advancing this agenda, including a multidisciplinary international expert panel. The series concluded that achieving this requires a comprehensive approach to addressing gaps in the ecosystem, including research and development (R&D), workforce development, technology transfer, regulatory systems, demand creation, and coordination. Strengthening R&D entails investment, capacity building, and equitable academic partnerships. A skilled workforce is essential, necessitating a coordinated approach through Regional Capability and Capacity Networks (RCCNs), training of vaccine manufacturing personnel, and academic programmes for sustainable workforce development. Technology transfer requires building trust between technology holders and recipients, alongside a supportive environment for knowledge exchange. Robust regulatory frameworks, including regional harmonization and strengthened National Regulatory Authorities (NRAs), are crucial for vaccine quality and safety, with the Africa Medicines Agency (AMA) providing oversight. Necessary market shaping through demand creation is achieved by advocating for procurement of locally produced vaccines, enhancing outreach for public trust, and operationalizing the African Pooled Procurement Mechanism (APPM). Coordination mechanisms are needed to optimize resource allocation, promote information sharing, and avoid redundancy. Strategic investments and policy support will be instrumental in achieving Africa’s vaccine manufacturing aspirations and long-term health security.

## Paper context

- **Main findings:** The gaps that must be addressed in order to build viable ecosystems for vaccine production and uptake in Africa include research and development, workforce development, technology transfer, regulatory systems, demand creation, and coordination.
- **Added knowledge:** Strengthening local vaccine manufacturing in Africa requires enhanced R&D, skilled workforce development, effective tech transfer, robust regulatory systems, demand creation, and coordinated agenda-setting.
- **Global health impact for policy and action:** The current analysis and recommendations – emanating from a seminar series hosted by Africa Centres for Disease Control and Prevention in collaboration with Karolinska Institutet and Charité Center for Global Health – could help realize Africa’s ambitions to become more self-reliant when it comes to vaccine development and manufacturing.

## Background

Vaccine shortages during the COVID-19 pandemic, the ongoing outbreak of mpox as well as the global funding shortfall for vaccines, underscore Africa’s urgent need for vaccine security and self-reliance. Recognizing this, a joint online seminar series was organized by Africa Centers for Disease Control and Prevention (CDC), the Karolinska Institutet, Stockholm and Charité Universitätsmedizin, Berlin, to discuss barriers and opportunities for advancing agenda including a multidisciplinary international expert panel.

## Main text

Africa was left far behind when COVID-19 hit close to 5 years ago, primarily due to low purchasing power, high-income countries (HICs) donation agreements not materializing [1] and stockpiling by HICs. Almost 2 years into the COVID-19 pandemic (November 2021), less than 0.5% of over 7 billion COVID-19 vaccine doses had been administered in low-income countries (LICs) [1]. Currently, we face a new large-scale epidemic; Mpox was declared as a public health emergency of continental security by the Africa CDC [2], and as a public health emergency of international concern by the World Health Organization (WHO) [3], on 13 and 14 August 2024, respectively. As of July 2025, 60 countries reported mpox cases, with the majority of cases and deaths occurring in Africa, among whom a large proportion were children. Unfortunately, the same trend continues, with disproportionate amounts of mpox vaccine doses to LICs that need them most [4]. From 5 to 7 November 2024, the WHO and Africa CDC met in Brazzaville, Congo, to discuss the urgent need for an estimated 18–22 million doses of mpox vaccines in the continent while, at the time of the meeting, only 280,000 doses of mpox vaccine had been deployed [4]. As of April 2025, 1 million mpox vaccine doses had been delivered to 10 African countries; however, only approximately 650,000 doses had been administered, with 90% of those in the Democratic Republic of the Congo (DRC) [5]. This stands in stark contrast to the rapid scale-up in access to COVID-19 vaccines globally [6], as at the same time, millions of doses of mpox vaccine (modified Vaccinia Ankara-Bavaria Nordic MVA-BN) were stored in HICs, primarily in Europe and USA [5].

The mpox outbreak has made it clear that the world remains as unprepared to share vaccines according to need as it was during the COVID-19 pandemic. Despite many good intentions, the global vaccine pool is not distributed in a manner that ensures equality and addresses the need to control outbreaks effectively.

The WHO has updated its Continental Response Plan to emphasize outbreak control and integration of mpox into routine health services. Diagnostic capacity has expanded markedly in DRC with more laboratories and the rollout of near-point-of-care tests [7]. However, over US$ 220 million is needed to close funding gaps for mpox alone [7], and the timing could not be worse. Since the Brazzaville meeting, new major challenges for global health security and pandemic preparedness have emerged, threatening global security. This includes a very significant reduction in humanitarian aid, not only through USAID accounting for 42% of all humanitarian aid but also from other HICs in Europe that are cutting their development aid budgets [8]. In June 2025, world leaders met at the Global Summit in Brussels to pledge support to Gavi, the Vaccine Alliance, for the new 5-year strategic period [9]. Although the numbers of donors were record high, multiple HICs prioritized their domestic budgets and pledges fell about 3 billion dollars short of the US$ 11.9 billion target budget needed to prevent millions of children from being unvaccinated in 2026–2030 [9].

This seriously undermines the global vaccine response by the main global actors such as the WHO, UNICEF, the Global Fund, and Gavi, reinforcing the importance of making Africa more self-sufficient when it comes to vaccine manufacturing. The ambitions are clear: Africa CDC among other actors have taken important initiatives to achieve improved vaccine security and self-reliance. The resulting Framework for Action (FFA) developed by the African Union [10] outlines the Platform for Harmonized African Health Products Manufacturing (PHAHM) towards building African capacity for vaccine research, development, production, and distribution to reach the target of 60% of vaccines to be manufactured on the continent by 2040.

What are the elements that must be in place to realize the goals set out by the African Union? Where are the voids in the ecosystem that must be filled by skill development and research, regulatory science, tech transfer, and bolstering vaccine demand?

Over a period of 5 months in 2024, a panel of multidisciplinary teams from Africa CDC, Charité Universitätsmedizin, Berlin, and Karolinska Institutet, Stockholm, ran a series of interactive online seminars to discuss the current status, review the plans, and propose interventions in the thematic areas considered essential for developing an ecosystem for sustainable vaccine production and uptake in Africa [11]. The speakers and panelists were selected from a diverse range of backgrounds and perspectives, including academia, government, industry, civil society, and international organizations. The target audience were researchers, policymakers, regulators, practitioners, donors, and civil society actors, and participation was open to all stakeholders. The content of the online sessions [11] remains freely available and was guided by the FFA across thematic areas considered essential for developing an ecosystem for sustainable vaccine production and uptake in Africa. Box 1 lists suggested interventions for strengthening local manufacturing in Africa that emanated from discussions during the vaccine seminar series discussions (Box 1).

**Box 1.**
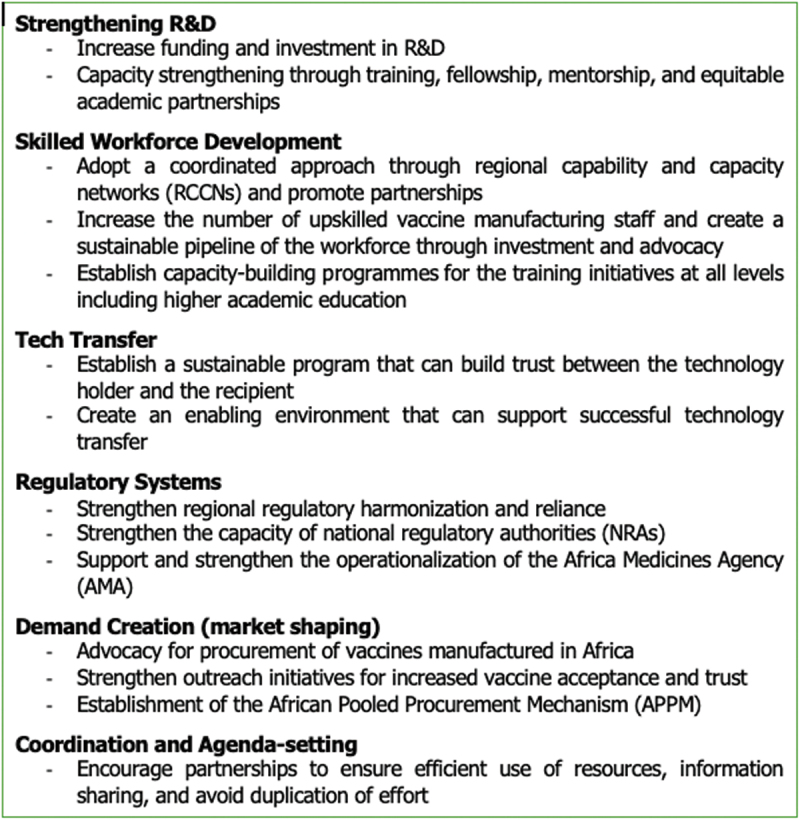
Suggested interventions for strengthening local manufacturing in Africa that emanated from discussions during the vaccine seminar series.

To successfully proceed from ‘skills to arms’, it will depend on an in-depth understanding of this complex ecosystem that must be in place on the African continent. To realize Africa CDC’s local vaccine manufacturing agenda by 2040, it is crucial to boost vaccine R&D and skills (train 12,500 full time employees in addition to today’s 3000 [12]), technology transfer, regulatory competence, and market-shaping initiatives such as pooled procurement mechanisms.

Academic institutions worldwide have a major role to play in this process. Building the vital competence and knowledge base requires training at all levels, from undergraduate to graduate education, as well as research on a number of critical issues, including vaccine development, quality assurance, and vaccine acceptance.

Equitable partnerships between universities in the Global South and North could help ensure that future virus outbreaks and pandemics can be mitigated by locally produced vaccines to avoid the unjust distribution of manufacturing capacity of essential health products. Investing in local manufacturing initiatives in Africa and the knowledge platform on which these must be built upon is key to ensuring self-sustained continental security and to close the technology divide that continues to haunt global health.

Sufficient financing is a cornerstone for all activities, including sustained commitment from African governments. Gavi’s African Vaccine Manufacturing Accelerator (AVMA), which aims to provide milestone-based financing to regional manufacturing and catalyse productive investment across the continent including technology transfer [9], is one example of the new financing models that will be required.

Recent studies reinforce and complement our main conclusions on the critical importance of investing in Africa’s scientific infrastructure, particularly in genomics and pathogen surveillance, as a foundation for health security [13,14]. These studies highlight the success of initiatives like the Africa Pathogen Genomics Initiative (Africa PGI), which rapidly scaled sequencing capacity during the COVID-19 pandemic [13,14]. This evidence aligns with the emphasis on R&D and workforce development as pillars of vaccine self-reliance and the need to retain talent, address the ‘brain drain,’ and build sustainable scientific ecosystems within Africa. Rubin Thompson et al. outlines the structural and financial requirements for building manufacturing hubs, including technology transfer, regulatory harmonization, and market incentives [14]. Together, this body of evidence complements our discussion of the PHAHM and the APPM and forms a coherent narrative that underscores the urgency and feasibility of Africa’s vaccine manufacturing ambitions.

## Data Availability

Not applicable. No primary data was used in this article.
